# Attitudes towards Doping and Related Experience in Spanish National Cycling Teams According to Different Olympic Disciplines

**DOI:** 10.1371/journal.pone.0070999

**Published:** 2013-08-21

**Authors:** Jaime Morente-Sánchez, Manuel Mateo-March, Mikel Zabala

**Affiliations:** 1 Faculty of Sport Sciences, University of Granada, Granada, Spain; 2 Spanish Cycling Federation, Madrid, Spain; 3 Miguel Hernández University, Elche, Spain; The Scripps Research Institute, United States of America

## Abstract

Attitudes towards doping are considered an influence of doping intentions. The aims of the present study were 1) to discover and compare the attitudes towards doping among Spanish national team cyclists from different Olympic disciplines, as well as 2) to get some complementary information that could better explain the context. The sample was comprised of seventy-two cyclists: mean age 19.67±4.72 years; 70.8% males (n = 51); from the different Olympic disciplines of Mountain bike -MTB- (n = 18), Bicycle Moto Cross -BMX- (n = 12), Track -TRA- (n = 9) and Road -ROA- (n = 33). Descriptive design was carried out using a validated scale (PEAS). To complement this, a qualitative open-ended questionnaire was used. Overall mean score (17–102) was 36.12±9.39. For different groups, the data were: MTB: 30.28±6.92; BMX: 42.46±10.74; TRA: 43.22±12.00; ROA: 34.91±6.62, respectively. In relation to overall score, significant differences were observed between MTB and BMX (p = 0.002) and between MTB and TRA (p = 0.003). For the open-ended qualitative questionnaire, the most mentioned word associated with “doping” was “cheating” (48.83% of total sample), with “responsible agents of doping” the word “doctor” (52,77%), and with the “main reason for the initiation in doping” the words “sport achievement” (45.83%). The major proposed solution was “doing more doping controls” (43.05%). Moreover, 48.67% stated that there was “a different treatment between cycling and other sports”. This study shows that Spanish national team cyclists from Olympic cycling disciplines, in general, are not tolerant in relation to doping. BMX and Track riders are a little more permissive towards the use of banned substances than MTB and Road. Results from the qualitative open-ended questionnaire showed interesting data in specific questions. These results empower the idea that, apart from maintaining doping controls and making them more efficient, anti-doping education programs are needed from the earliest ages.

## Introduction

The use of performance enhancements has been a problem in competitive sport for decades [1]. Bloodworth et al. [2] mentioned that the use of doping substances in cycling appeared in the 1890s, when cyclists were given substances such as extra caffeine, strychnine and even cocaine to improve their performance. More recently, the Festina case scandal in Tour de France of 1998 provided evidence of a systemic doping problem in sport [2], [3]. With the purpose of preventing it, the phenomenon of doping in sport has been studied by medical, physiological and social science researchers in recent years [4].

For Lucidi et al. [5], “attitudes” were the strongest predictors of intention to use banned substances. Nevertheless, a recent work published by Barkoukis et al. [6] argues that doping intentions are influenced by a) Distal influences (self-determination, sportpersonship orientations, and achievement goals), and b) Proximal influences (situational temptation and perceived behavioral control, descriptive and subjective norms, and attitudes). Distal influences have an indirect effect on proximal influences, and the latest have a direct influence on doping intentions. So, attitudes seem to play an interesting role in doping intentions, as achievement goals in sports and sportpersonship beliefs seem to influence doping intentions indirectly through the effects of attitudes and self-efficacy beliefs.

Other previous studies also related attitudes a) to achievement goal orientations [7], b) to situational temptation [8], c) to doping intention itself [9], or d) to knowledge, behaviors and education [10].

In relation to the type of tools employed to assess attitudes towards doping in sport in the scientific literature, just a few used validated tools but not in elite athletes [1]. Although past evidence used “non-validated” instruments, these were based on well-established theories and got very useful information. The advantage of using a standard validated questionnaire is mainly that different contexts (sports, countries, gender or age) could be better compared. By the way, it has been suggested the use of both non-validated and validated tools (qualitative and quantitative) and, ideally including less-invasive biomedical tests [1].

Most of the studies related to attitudes towards doping in elite sport used samples of a mix of athletes from different disciplines or analyzed big samples from team sports, so that there exists a relative dearth of scientific research in relation to our object of study, doping and elite cycling, probably the most persecuted sport [1]. According to Lentillon-Kaestner et al. [3], in top-performing cycling the use of PED was endemic among the cycling teams to the extent that it became institutionalized [11]–[13] and was quasi-tolerated by the professional cycling community [14] before the Festina scandal in 1998. They also concluded that the use of banned substances is less widespread nowadays. In Spain, after infamous and unfortunate scandals like “Operación Puerto” in 2006 or the Armstrong case in 2012, it has been suggested that studies of this type about doping in sport, and more concretely focused on elite cycling and doping, are necessary [1].

Considering the international view about the phenomenon of doping in Spanish cycling and taking into account the lack of relevant related studies, we have undertaken an investigation of Spanish elite cyclists’ attitudes (using psychometric testing) and experiences (by means of some open-ended questions related to the context).

The reasons why we consider this study as very important are 1) because Spain has been a reference for cycling all over the world due to the big sporting success in the past, especially in road cycling (2006–2009 tour de France consecutive wins −2010 winner was dispossessed for doping reasons-, gold medal in road men cycling at Beijing 2008, etc.), and 2) the scandal of Puerto case in Spain involved an important number of cyclists and other athletes from this and other countries. So, the aims of the present study were 1) to discover and compare the attitudes towards doping among Spanish national team cyclists from different Olympic disciplines, as well as 2) to get some complementary information that could better explain the context.

## Methods

### Sample

A total of 72 Spanish national team cyclists (mean age: 19.67±4.72 years) participated in the study. The gender distribution was 70.8% males (n = 51) and 29.2% females (n = 21). The total sample was divided into four groups according to the 4 different Olympic cycling disciplines: Mountain bike -MTB- (n = 18; mean age: 17.6±2.53 years; age range: 16–26 years; 83.3% men), Bicycle Moto Cross -BMX- (n = 12; mean age: 19.1±3.89 years; age range: 16–28 years; 100% men), Track -TRA- (n = 9; mean age: 27.67±6.18 years; age range: 22–38 years; 77.8% men) and Road -ROA- (n = 33; mean age: 18.62±2.82 years, age range: 16–30 years; 54.5% men). The high range of age in the samples is because the national Federation usually combine in training camps riders of different ages: those that will participate in the next Olympics and those who would be potentially Olympic athletes in the near future who in that moment are competing at the highest level in lower categories like Junior or Under 23 (world and European championships). All of the participants of this study belonged to the Spanish National cycling team and consequently had competed previously in international championships (the European championship, World Cup, and, for some of them, previous Olympic Games). Additionally, 11 participants of this study were among the 17 cyclists representing Spain who participated in the London 2012 Summer Olympics. Therefore, all were considered elite athletes. The testing protocol and data handling were approved by the Ethics Committee of the University of Granada (Spain).

### Measures

A cross-sectional descriptive design was carried out by means of a validated questionnaire, as well as using a bespoke open-ended questionnaire to better explain the context. Performance Enhancement Attitude Scale (PEAS) [4]. The PEAS is a 17-question 6-point Likert-type scale, with points anchored from strongly disagree (1) through disagree (2), slightly disagree (3), slightly agree (4), agree (5) to strongly agree (6). No neutral response if offered and all 17 items are scored in the same direction. The overall scores range from 17 to 102 points (giving a theoretical middle-point of 59.5), with higher scores representing a more lenient attitude toward doping. This scale has been used in previous studies and has shown good psychometric properties [4], [15]. Although its satisfactory validation in Spanish is still in publication process, we found Cronbach Alpha values ranging from 0.70 to 0.84 among all the groups studied. Participation was completely voluntary. To provide the participants with a sense of security, and thus to obtain reliable data, the principle of anonymity was secured.

To complement the PEAS, a qualitative open-ended questionnaire about the athletes’ own experience and opinion was used. This questionnaire, used in other previous studies [16]–[18], was considered to better understand the context of the PEAS values. Athletes were asked to respond to seven questions delving into the reasons for doping in professional cycling: 1) words associated with doping; 2) agents responsible for doping; 3) differences between cycling and other sports; 4) reasons for initiation into doping; 5) has doping been suggested to you?; 6) have you seen another person inciting or being incited to dope?; and, finally, 7) proposed solutions.

For the purpose of this study, “doping”, “drugs” or “banned substances” were considered as those substances that are prohibited by the WADA or other governing body in training and/or sport competition, and this was explained to participants before responding.

### Data collection

Participants were recruited via personal and professional contacts in their national team training camps prior to the London 2012 Olympics Games. After the participants gave written informed consent, the anonymous questionnaires were self-administered. Written informed consent was obtained from parents or guardians on the behalf of minors involved in the study. There was no time limit for completing them. A regular coding system was used by the research assistant and the data were submitted in Excel files.

### Analyses

Data characteristics were shown as frequencies, percentages, means, and standard deviations. For the PEAS, the Kolmogorov-Smirnov Test was applied to ensure a Gaussian distribution of the results, followed by the Levene test to verify the homogeneity of variance. Then, when we noted that the results followed a non-normal distribution, a non-parametric analysis was conducted. The Mann-Whitney U-test, using Bonferroni post-hoc correction, was carried out (critical statistical significance: p<0.0125). Statistical analyses were performed using IBM-SPSS 20.0 software.

## Results

### PEAS - Performance Enhancement Attitude Scale

In general, the mean overall score (17–102) was 36.12±9.39. Taking the different analyzed groups into account, overall scores were, respectively: MTB: 30.28±6.92; BMX: 42.46±10.74; TRA: 43.22±12.00; ROA: 34.91±6.62. Regarding overall scores (*see* Table 1), significant differences were observed between MTB and BMX (p = 0.002) and between MTB and TRA (p = 0.003).

**Table 1:** Descriptive statistics and comparisons between different Olympic cycling disciplines (Total sample, Road, MTB, BMX and Track) for the overall score of the Performance Enhancement Attitudes Scale (PEAS).

	Total sample (n = 72) Mean (SD)	MTB team (n = 18) Mean (SD)	BMX team (n = 12) Mean (SD)	Track team (n = 9) Mean (SD)	Road team (n = 33) Mean (SD)	p*
**PEAS Overall Score**	36.12 (9.39)	30.28 (6.92)	42.46 (10.74)	43.22 (12.00)	34.91 (6.62)	p = 0.002^1–2^; p = 0.003^1–3^

* p≤0.0125.

1–2 Significant differences between MTB and BMX groups.

1–3 Significant differences between MTB and Track groups.

### Open-ended qualitative questionnaire

**Figure 1:**
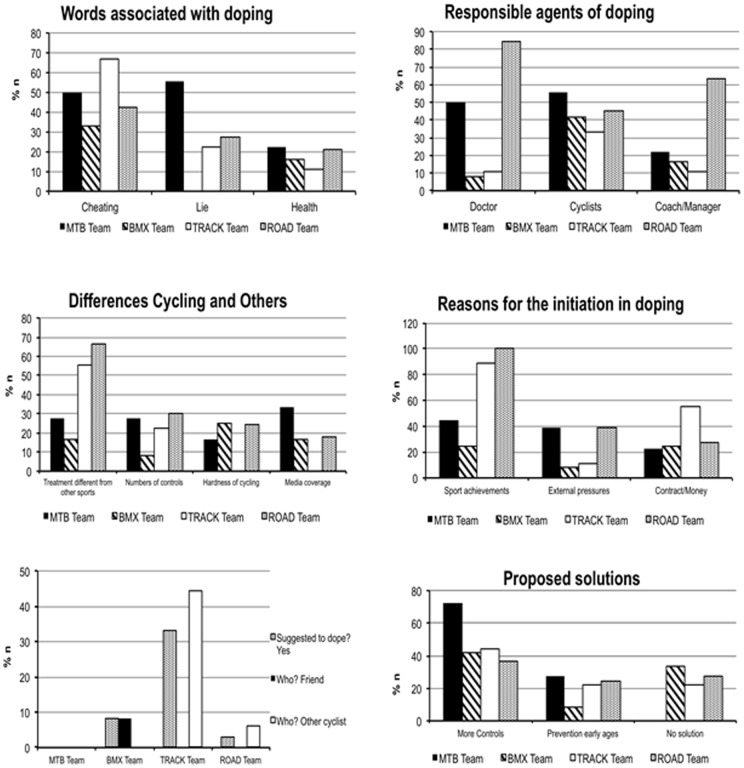
Percentage of participants who make a specific statement (% n).

The data obtained were expressed in terms of percentage of participants who make a specific statement (% n). Participants did not have a limit on their number of possible answers, and consequently the sum of the percentages is not adjusted to 100%. Different groups (Total sample, BMX, MTB, TRA and ROA) were compared (see Table 2 and Figure 1):

**Table 2:** Descriptive statistics (percentage of total sample of each group −% n−), for the different Olympic cycling disciplines (Total sample MTB, BMX, Track and Road).

	%Total sample (n = 72)	%MTB Team (n = 18)	%BMX Team (n = 12)	%Track Team (n = 9)	%Road Team (n = 33)
**Words associated with doping** *					
Cheating	45.83	50.00	33.33	66.67	42.42
Lie	29.16	55.56	0	22.22	27.27
Health	12.50	22.22	16.17	11.11	21.21
**Responsible agents of doping** *					
Doctor	52.77	50.00	8.33	11.11	84.85
Cyclists	50.00	55.56	41.67	33.33	45.45
Coach/Manager	41.66	22.22	16.67	11.11	63.64
**Differences Cycling and Other sports** *					
Treatment different from other sports	48.67	27.78	16.67	55.56	66.67
Numbers of controls	20.83	27.78	8.33	22.22	30.30
Hardness of cycling	19.44	16.67	25.00	0	24.24
Media coverage	19.44	33.33	16.67	0	18.18
**Reasons for the initiation in doping** *					
Sport achievements	45.83	44.44	25.00	88.89	100
External pressures	29.16	38.89	8.33	11.11	39.39
Contract/Money	26.38	22.22	25.00	55.56	27.27
**Have you been suggested to dope?**					
Yes	6.94	0	8.33	33.33	3.03
**Who?**					
Friend	1.39	0	8.33	0	3.03
Other cyclist	5.56	0	0	33.33	0
**Have you seen other people inciting others or being incited?**					
Yes	9.72	0	8.33	44.44	6.06
**Who?**					
Friend	1.39	0	8.33	0	0
Other cyclist	5.56	0	0	44.44	6.06
**Proposed solutions** *					
More Controls	43.05	72.22	41.67	44.44	36.36
Prevention early ages	22.22	27.78	8.33	22.22	24.24
No solution	20.83	0	33.33	22.22	27.27

* Each participant could mention as many answers as needed, but just the most relevant are presented (the rest ranged from 1.39 to 5.56% are not considered).

1. Words associated with doping: the three most mentioned expressions were “cheating” (% n: 48.83), “lie” (% n: 29.16), and “healthy” (% n: 12.5). In the group comparison, the most repeated words were: MTB (“lie”; % n: 55.56), BMX (“cheating”; % n: 33.33), TRA (“cheating”; % n: 66.67) and ROA (“cheating”; % n: 42.42).
2. Agents responsible for doping: the three most mentioned agents were “doctor” (% n: 52.77), “cyclists” (% n: 50.00), and “coach/manager” (% n: 41.66). In relation to different analyzed groups, the most suggested agents responsible of doping were: MTB (“cyclists”; % n: 56.56), BMX (“cyclists”; % n: 41.67), TRA (“cyclists”; % n: 33.33) and ROA (“doctor”; % n: 84.85).
3. Differences between Cycling and Other sports: the four most mentioned differences were “cycling receives a different treatment in comparison with other sports” (% n: 48.67), “numbers of controls” (% n: 20.83); “hardness of cycling” and “media coverage” (% n: 19.44). For different groups, the most mentioned differences were: MTB (“media coverage”; % n: 33.33), BMX (“hardness of cycling”; n: 25.00), TRA (“cycling receives a different treatment in comparison with other sports”; % n: 55.56) and ROA (“cycling receives a different treatment in comparison with other sports”; % n: 66.67).
4. Reasons for the initiation of doping: the three most mentioned reasons were “sport achievements” (% n: 45.83), “external pressures” (% n: 29.16), and “contract/money” (% n: 26.38). Taking into account the different groups, the most repeated reason was “sport achievements”: MTB (“% n: 44.44), BMX (% n: 25.00), TRA (% n: 88.89), and ROA (% n: 100.00). For BMX riders, the previous option was mentioned as frequently as “Contract/Money” (% n: 25.00).
5. Has doping been suggested to you?: five riders of the total sample stated “yes” (5/72, 6.94%). In relation to different groups, respectively: MTB (nobody), BMX (1/12, 8.33%), TRA (3/9, 33.33%), and ROA (1/33, 3.03%). Riders from the TRA and ROA groups were suggested by “another cyclist” while the rider from BMX was recommended by a friend.
6. Have you ever seen other people inciting others or being incited?: seven riders of the total sample stated “yes” (7/72, 9.72%). The breakdown by different groups was: MTB (nobody), BMX (1/12, 8.33%), TRA (4/9, 44.44%), and ROA (2/33, 6.06%). The four riders from TRA group were suggested by “another cyclist” while the rider from BMX was recommended by a friend.
7. Proposed solutions: the three most mentioned suggestions to eradicate doping in sport were “more controls” (% n: 43.05), “prevention at early ages” (% n: 22.22), and “no solution” (% n: 20.83). For different groups, the most suggested solution was “more controls”: MTB (% n: 72.22), BMX (% n: 41.67), TRA (% n: 44.44), and ROA (% n: 36.36).

## General Discussion

The results of the present study showed that the cyclists of the Spanish national teams of cycling are generally not tolerant in relation to doping. However, BMX and Track riders were a little more permissive towards the use of banned substances than MTB and Road. In addition, results from the open-ended qualitative questionnaire have shown interesting and specific data (e.g., reasons for use or responsible agents), which should be taken into account. Regarding those potentially dangerous groups, it could be interesting to analyze them more exhaustively to look for the causes of that certain permissiveness, to intervene more effectively. These findings empower the idea that, apart from more efficient controls, anti-doping education programs are needed from early ages.

According to the systematic review carried out by Morente-Sánchez and Zabala [1], there were no previous specific studies that assessed attitudes towards doping in elite athletes by means of this validated scale. According to Petróczi and Aidman [4], demonstrated reliability and validity were poor and inferences could not be made in the majority of the studies in this field. One of the few studies that have used this PEAS (the higher the score, the more permissive the attitude towards doping shown) was developed by Uvacsek et al. [15]. In this study, among 82 Hungarian competitive (non-elite) athletes assessed, confessed doping users (12%) scored, as expected, significantly higher on the PEAS (p<0.05) when compared with those who reported no use of banned drugs (46.8±13.32 and 34.43±8.74, respectively). Likewise, in another study [19], with 2022 amateur cyclists as a sample (confessed users  = 164; non-users  = 1858), overall scores were, respectively: 48.87±15.98 and 40.98±11.95. Petróczi and Aidman [4] analyzed several samples such as elite athletes from Hungary (n = 102; confessed users  = 5; non-users  = 97), obtaining the following scores, respectively (39.20±17.54 vs. 35.85±10.12). According to the present study, in general, Spanish cyclists of the national teams are against of doping, though BMX and Track riders are more permissive towards PED use than MTB and Road. The case of track cyclists is especially risky because this sample is the oldest and, as a result, their attitudes and beliefs should be stronger as a consequence of their larger experience. It is interesting that MTB cyclists, the youngest group, showed a very low score, which could mean that new generations are more aware of doping. As a practical application, we could consider that for those more permissive groups, whose scores are close to those of confessed users, a deep analysis and monitoring of this sample appears necessary.

Conversely, ad-hoc questionnaires allow getting more specific information by using direct questions in different perspectives related to this topic like “words associated to doping”, “reasons for use”, or “agent responsible”. For instance, it has been shown that, for all the Olympic cycling disciplines, the word most associated with doping was “cheating” (% n: 48.83), except for the BMX team mentioning a related term (“lie”, % n: 55.56). It is remarkable that terms like “performance” or “win” did not appear in the first positions in the order of the most mentioned answers.

Regarding agents responsible for doping, similar open-ended questionnaire was used with a sample of 87 Spanish cycling team managers who recognized themselves like the main responsible [16], [17]. In a similar study, Zabala et al. [18] stated that for professional cyclists the main responsible agents that evoke doping were 1) Team Managers, 2) Doctors, and 3) the cyclists, while for the team managers the responsible were the 1) pressure of sponsors, 2) cyclists,3) team managers, and 4) doctors. Nieper [20] in a survey of 34 British junior team athletes noted that coaches provided the greatest influence (65%), followed by sports dieticians (30%) and doctors (25%). By contrast, Somerville et al. [21] reported that the doctor was the first option for 62% (46/74) of athletes in their study. Conversely, in this study, following Lentillon-Kaestner et al. [22], who stated that the pressure of team staff and doctors on cyclists’ use of banned substances has become less important and direct after the latest doping scandals, three of the four groups (MTB, BMX and Track) recognized themselves, “cyclists”, as the main agents responsible for doping. Therefore, it seems essential to raise awareness and re-educate both professional groups (doctors and coaches) in addition to cyclists, because of their recognized and important influence on athletes. In line with this conclusion, other authors show how sport should change according to the so-called “athlete 2.0” concept as a collaborative challenge [23].

In relation to the reasons for initiation of doping, Lentillon-Kaestner and Carstairs [24] interviewed 8 young elite cyclists who admitted that they were open to using doping substances themselves if it was the key to continuing their cycling career, but only after they became professional. Similar results were stated by Backhouse et al. [10] in their extensive review to WADA in 2007. The results of our study are relevant to this since “sport achievements” was the most mentioned motive for all the groups, though BMX riders also considered “contract/money” at the same level of importance as an inducement to doping behavior. In the same way, Striegel et al. [25], studying the prevalence of doping in 978 German elite athletes, reported that the most repeated reasons for drug use were to achieve athletic success (86%) and for financial gain (74%). In addition, in another study (n = 40), various factors were acknowledged as potential reasons for use, most notably injury recovery and the economic pressures of elite sport [26]. Moreover, when cyclists were asked about the differences in relation to doping treatment between cycling and other sports, in general, they strongly highlighted the existence of “different treatment to other sports” (% n: 48.67). In this sense, different treatments among different types of sports in relation to doping have been studied in several investigations. English professional footballers were tested for drugs less often than many other elite athletes, only about 33% per year, according to Waddington et al. [27]. It could be argued that differences between sports could be related to the independence that sport federations have in this respect in most competitions, and this possible difference seems to be reduced only in the Olympic Games. In this sense, Spanish elite cyclists’ opinions are in accordance with other studies that also consider that there is a different treatment in the quantity and quality of drug testing among sports [28].

Focusing on the direct questions such as “Has doping ever been suggested to you” or “Have you ever seen other people inciting others or being incited?", it was observed that 5/72 (6.94%) and 7/72 (9.72%) riders answered “yes”, respectively, for each question. The Track rider’s group, despite being a small sample, reported a high percentage of affirmative answers (3/9, 33.3%; and 4/9, 44.4%, respectively). When this result is added to their mean age and their PEAS score, this makes the Track group at greater risk in relation to doping. The percentage of “yes” for those questions was higher (approximately 50%) in a study that involved 87 Spanish cycling team managers [16], [17]. In addition, in reviewing the latest scientific literature in this field on attitudes towards doping in elite athletes, it is interesting to observe the emergence of a concept of so-called “false consensus effect” [15], [29], which suggests that athletes who have a history of PED use overestimate the prevalence of drug use among other athletes. Tangen and Breivik [30] also showed that an individual's decision to take banned substances is influenced by the assumption that his or her competitors are also taking drugs [31]. Therefore, it seems clear that if athletes believe that others are taking doping substances, this can push some of them to start using them as well, and this could be like a vicious circle that feeds the pro-doping culture.

Finally, regarding proposed solutions when they were asked about what they would do to eradicate the phenomenon of doping in sport, it was interesting to observe the pessimistic point of view of Spanish national team cyclists since 20.83% of them stated that this problem had no solution. The most proposed option for all the groups was “more controls”(% n: 43.05). In spite of this, increasing drug testing is not synonymous with success in relation to doping prevention. According to Alaranta et al. [28], “controlling doping only by tests is not sufficient; a profound change in the attitudes, which should be monitored repeatedly, is needed”. This statement summarizes the current situation in relation to doping in sport in accordance with most of the studies reviewed. According to Peters et al. [32] and Lentillon-Kaestner et al. [22], in the fight against doping, preventive measures are necessary to establish and fortify attitudes towards doping at an early stage. We encourage institutions to invest more money by balancing the costs of controls and prevention programs from early ages as suggested by Morente-Sánchez and Zabala [1]. Controls are obviously needed, as are more effective educational programs that do not require large investments.

This study is not free of limitations since work based on questionnaires covering a banned practice has limits: answers may be deliberately false as the participants questioned may not wish to reveal that they or their teammates use PED, even if anonymity and confidentiality are guaranteed by the researchers. However, a bigger sample size could be more representative although the quality of the selected participants is high: the Spanish National Team cyclists of the four Olympic disciplines (MTB, BMX, track and road).

Taking everything into account, we suggest that descriptive studies to design effective intervention programs should be carried out by means of the same tools. For this purpose, the PEAS could be used as a standard measurement instrument to assess attitudes towards doping so that data are more reliable and valid, and practical applications can be developed efficiently, even when complemented with other tools such as interviews or ideally biomedical tests. Focusing on cycling in particular, we consider, after the most recent media doping cases (such as Puerto or Armstrong), that now is the ideal moment to establish a cooperative structure among the interested parties [most importantly, cycling events organizations (Tour, Giro and Vuelta), the International Cycling Union (ICU), the World Anti-Doping Agency (WADA), and the world of sport science research] to analyze the current situation deeply and subsequently to design specific programs and other activities for prevention and to fight against the phenomenon of doping.

## Conclusions

The main conclusion of this study is that the Spanish national team cyclists of the different Olympic disciplines in general are not tolerant of doping. However, BMX and Track riders appear more permissive towards the use of banned substances than MTB and Road. Additionally, results from an open-ended qualitative questionnaire have shown interesting and specific data (e.g., reasons for use or responsible agents), which should be taken into account. Regarding those potentially dangerous groups, it could be interesting to analyze them more exhaustively looking for the causes of that certain permissiveness to intervene more effectively. This emphasizes the idea that, apart from maintaining doping controls and making them more efficient, anti-doping education programs are needed from the earliest ages, focusing not only on athletes but also on their context-doctors, coaches, and family.
